# Vitamin D Deficiency Is Not Associated with Diabetic Retinopathy or Maculopathy

**DOI:** 10.1155/2016/6156217

**Published:** 2016-01-14

**Authors:** Uazman Alam, Yasar Amjad, Anges Wan Shan Chan, Omar Asghar, Ioannis N. Petropoulos, Rayaz A. Malik

**Affiliations:** ^1^Centre for Endocrinology and Diabetes, Institute of Human Development, University of Manchester and the Manchester Royal Infirmary, Central Manchester Hospital Foundation Trust, Manchester M13 9NT, UK; ^2^Department of Medicine, Barts and the London School of Medicine and Dentistry, London E1 2AD, UK; ^3^Weill Cornell Medical College in Qatar, Doha, Qatar

## Abstract

*Background*. Experimental and clinical studies suggest a possible association between vitamin D deficiency and both diabetic retinopathy and maculopathy.* Methods*. We have performed a cross-sectional study in adults with types 1 and 2 diabetes mellitus. The relationship between the presence and severity of diabetic retinopathy and maculopathy with serum 25-hydroxyvitamin D concentration was evaluated using logistic regression analyses in the presence of demographic and clinical covariates.* Results*. 657 adults with diabetes were stratified based on retinopathy grading: No Diabetic Retinopathy (39%), Background Diabetic Retinopathy (37%), Preproliferative Diabetic Retinopathy (21%), and Proliferative Diabetic Retinopathy (3%), respectively. There were no differences in serum 25-hydroxyvitamin D concentrations (25(OH)D) between the groups (15.3 ± 9.0 versus 16.4 ± 10.5 versus 15.9 ± 10.4 versus 15.7 ± 8.5 ng/mL, *P* = NS). Logistic regression analysis demonstrated no statistically significant relationship between the severity of retinopathy and serum 25(OH)D. Furthermore, there was no difference in serum 25(OH)D between those with (*n* = 94, 14%) and those without (*n* = 563, 86%) Diabetic Maculopathy (16.2 ± 10.0 versus 15.8 ± 9.8, *P* = NS) and no relationship was demonstrated by logistic regression analyses between the two variables.* Conclusions*. This study has found no association between serum 25(OH)D and the presence and severity of diabetic retinopathy or maculopathy.

## 1. Introduction

The prevalence of diabetic retinopathy (DR) approaches 93 million people worldwide [1] and is one of the leading causes of premature visual loss in the UK and worldwide [2]. Indeed, the World Health Organization estimates that whilst diabetic retinopathy accounts for approximately 5% of the global prevalence of blindness, the prevalence rises sharply to 15–17% in developed countries [3]. Several risk factors are implicated in the aetiology of DR with hyperglycemia and hypertension showing the strongest association [4], yet interventions aimed at correcting these risk factors have demonstrated moderate success [5, 6]. Therefore, the interactions between neural and retinal vascular dysfunction and the mechanisms resulting in retinal pathology including neovascularisation have been questioned recently [7]. Furthermore, micronutrients including vitamin C, vitamin E, and magnesium have been postulated to play a role in DR [8].

Vitamin D deficiency has been linked to a host of cardiovascular diseases including diabetes and hypertension [9, 10]. Vitamin D receptor (VDR) genotypes have been associated with the cumulative prevalence of diabetic retinopathy [11]. In two separate studies of the VDR gene in the French population, FokI and TaqI single nucleotide polymorphisms have been associated with DR [12, 13]. In a study of Caucasians with C-peptide-negative type 1 diabetes, there was a novel association between the functional* Fok*I VDR polymorphism and severe DR [12]. VDR dependent calcium binding proteins have been isolated in the human retina, particularly in the photoreceptor layer of the cones [14], and immunostaining in animal models has shown that VDR is expressed in the ganglion cells, the inner and outer plexiform layer, and the photoreceptor layer [15]. In an in vitro study of retinoblastoma tissue expressing VDR, supplementation with vitamin D resulted in a reduction of growth and apoptosis of the retinoblastoma cells [16]. 1,25-Dihydroxyvitamin D_3_ (1,25(OH)_2_D_3_) closely regulates Vascular Endothelial Growth Factor in experimental models [17] and there is an inverse correlation of 25(OH)D with Vascular Endothelial Growth Factor, postulated to be related to tissue hypoxia [18]. In a mouse model of ischaemic retinopathy, 1,25(OH)_2_D_3_ was shown to inhibit neovascularisation in retinal tissue [19]. Vitamin D may also have a direct effect on the renin-angiotensin-aldosterone-system and the renin-angiotensin-aldosterone-system is known to be overexpressed in patients with type 1 diabetes and retinopathy [20] and blockade of this system reduces DR progression [21]. A Vitamin D analogue (paricalcitol) has shown an improvement in microalbuminuria through a mechanism related to inhibition of renin-angiotensin-aldosterone-system [22].

Aksoy et al. demonstrated an inverse correlation in a Turkish cohort between worsening diabetic retinopathy and lower 1,25-dihydroxyvitamin D_3_ (active vitamin D) in a population of 66 subjects [23]. Furthermore, severe vitamin D deficiency has been shown to predict not only mortality but the development of nephropathy and retinopathy in type 1 diabetes mellitus [4]. In a recent cross-sectional study of children and adolescents with type 1 diabetes, retinopathy prevalence was higher in children and adolescents with lower levels of vitamin D [24]. Other cross-sectional studies which have assessed vitamin D status in relation to DR in adults either have had small numbers [25] or have been based on retrospective analysis of data collected from the National Health and Nutrition Examination Survey between 1988 and 1994 [26]. However, since then, the targets for glycaemia, blood pressure, and lipids have changed and also this study made no assessment of Diabetic Maculopathy [26]. Therefore, we have undertaken a study to establish the relationship between vitamin D status and the severity of DR and maculopathy in a large adult population with type 1 and type 2 diabetes.

## 2. Method

All patients attending clinics were assessed for the level of 25(OH)D, irrespective of a history suggestive of vitamin D deficiency. Written informed consent and ethical approval were not required as the data were extracted retrospectively and did not extend beyond standard clinical practice. All patient records and information were anonymised and deidentified prior to analysis. 25(OH)D was added as a standard routine test from June 2009 due to the high levels of deficiency noted. This was a retrospective analysis of data which had been collected already in our clinic for clinical rather than research reasons; that is, the patients with diabetes attending clinic underwent assessment of vitamin D as the clinical practice was to assess vitamin D in all patients and subsequently treat those who are deficient. These same patients were also undergoing retinal assessment as part of the annual review under the English retinal screening programme. The data (vitamin D and retinopathy grade) were not collected specifically for this analysis. There were a sample of 657 subjects in this retrospective study and prospective sample size analyses were inappropriate as all data available were assessed.

## 3. Subjects

All participants were aged ≥ 18 years attending clinics at the Central Manchester Foundation Trust, Manchester, and the assessment was conducted from August 2009 to May 2011. Those with renal impairment (eGFR <30 mL/min/1.73 m^2^ (CKD stages 4 and 5)), granulomatous diseases (tuberculosis, sarcoidosis, etc.), and malabsorption syndromes (coeliac disease, bacterial overgrowth, and concomitant orlistat treatment), pregnant and lactating women, and those currently on vitamin D supplementation were excluded from the analysis. Biases were limited by using an unselected cohort of subjects, not based on symptomatology of vitamin D deficiency.

### 3.1. Blood Pressure and Anthropometric Measurements

Body Mass Index (BMI) was measured as per the standard equation mass (kg)/(height(m))^2^. Weight was measured with a digital scale (Seca 701, Seca, Hamburg, Germany) to the nearest 0.1 kg and height was measured to the nearest 0.1 cm. Blood pressure measurements were obtained with the use of an automated device (Dinamap pro 100v2, GE Medical Systems, Freiburg, Germany) with an appropriate cuff size. A minimum of two measurements of systolic and diastolic blood pressures were made five minutes apart with the lowest reading recorded and the mean of the preceding 2-year blood pressure results was used. Metabolic variables were also recorded with a mean of 2-year retrospective readings for glycosylated haemoglobin A1c (HbA1c) and components of the lipid profile (total cholesterol (CHL), high-density lipoprotein cholesterol [HDL-C], and triglycerides). The following measurements were taken as “spot readings” at the same date as baseline 25(OH)D measurements: Body Mass Index (BMI), bone profile markers such as corrected calcium (CCa), alkaline phosphatase (ALP), and estimated glomerular filtration rate (eGFR).

### 3.2. Assessment of Demographics, Cardiovascular Disease, and Medications

An assessment of patient demographics, previous cardiovascular events, and medications were made through analysis of medical records and an in-hospital medical record database (Diamond database, Hicom, Surrey, UK). Subject demographics extracted were age, sex, ethnicity (Caucasian, South Asian, Far East Asian, and Afro-Caribbean descent), smoking status (never, previous, and current), and type (types 1 and 2 diabetes) and duration of diabetes. Dates of baseline 25(OH)D were used to obtain respective retinopathy screening data. Only retinopathy screening data within 1 year of the baseline 25(OH)D and prior to vitamin D supplementation were included.

Baseline 25(OH)D status and retinopathy data were collected for 657 patients who had attended their retinopathy screening appointments. The retinopathy data were collected according to the grading criteria of the National Screening Committee [27]. Previous studies have shown acceptable level of quality and accuracy of grading compared to expert graders within the English National Screening Committee [28]. The national guidelines do not contain R1.5 or M0.5 grades and are categorised as Preproliferative Diabetic Retinopathy and Diabetic Maculopathy, respectively. These subgradings were used locally in screening centres and have been included. Retinopathy was graded as follows:

 
*R0*: No Diabetic Retinopathy (NDR).
 
*R1*: Background Diabetic Retinopathy (BDR): microaneurysms, retinal haemorrhages, and exudates.
 
*R1.5*: moderate numbers of intraretinal haemorrhages, hard exudates >1 disc diameter (DD) from fovea, and 3–6 cotton wool spots visible.
 
*R2*: Preproliferative Diabetic Retinopathy (PPDR): venous beading or looping, deep haemorrhages visible, and other microvascular anomalies visible.
 
*R3*: Proliferative Diabetic Retinopathy (PDR): new vessel formation, vitreous haemorrhage, preretinal haemorrhage or fibrosis, and/or retinal detachment.
 
*M0*: no maculopathy.
 
*M0.5*: hard exudates within the arcades >1DD from the centre of the fovea.
 
*M1*: exudates <1DD from the centre of the fovea; retinal thickening <1DD from the centre of the fovea.
 
*P0*: no photocoagulation scarring.
 
*P1*: photocoagulation scarring.

### 3.3. 25(OH)D Assay

Serum was separated from whole blood and stored at −20°C until assay. The assay used was an automated platform assay (ImmunoDiagnostic Systems Ltd., Bolden, Tyne and Wear, UK) and is based on chemiluminescence technology. The assay was performed exactly as per the manufacturer's instructions. Briefly, samples were subjected to a pretreatment step to denature the vitamin D binding protein. The treated samples were then neutralised in assay buffer and a specific anti-25(OH)D antibody labelled with acridinium was added. Following an incubation step, magnetic particles linked to 25(OH)D were added. Following a further incubation step, the magnetic particles were “captured” using a magnet. After a washing step and addition of trigger reagents, the light emitted by the acridinium label was inversely proportional to the concentration of 25(OH)D in the original sample. Concentration of 25(OH)D was calculated automatically using a 4-point logistic curve. The reportable range of the assay was 5–140 ng/mL. Inter- and intra-assay variation of the in-house control were 5.6% and 9.7%, respectively.

### 3.4. Statistical Analysis

Data were analysed using StatsDirect (StatsDirect, Altringham, Cheshire, UK). The data were stratified according to retinopathy (NDR, BDR, PPDR, and PDR) and maculopathy (no maculopathy and maculopathy) status and a comparison of means was undertaken using either ANOVA or Krus-Kal Wallis for DR data and Unpaired *t*-test or Mann-Whitney *U* test for maculopathy data. Chi-squared test was used for aetiology of diabetes, ethnicity, gender, and smoking status. Logistic regression analyses were undertaken to assess the association between serum 25(OH)D levels and retinopathy and maculopathy status (either present (1) or not present (0)), adjusting for mean values of duration of diabetes, smoking status, HbA1c, total cholesterol, HDL, triglycerides, and systolic and diastolic blood pressure. Further assessment of the results comparing vitamin D categories (severely deficient (<10 ng/mL), deficient (10–<20 ng/mL), insufficient (20–<30 ng/mL), and sufficient (>30 ng/mL)) and retinopathy (NDR, BDR, PPDR, and PDR), maculopathy (no maculopathy and maculopathy), and photocoagulation status (no photocoagulation and photocoagulation) was performed using Chi-squared testing. Appropriate statistical analyses were employed depending on the normality of the data. Overall, the *P* value was maintained at 0.05 for multiple comparison tests (Bonferoni adjustment or Dwass-Steel-Chritchlow-Fligner pairwise comparison). Statistically significant results were deemed at a *P* value ≤0.05.

## 4. Results

657 subjects were stratified according to their retinopathy status: No Diabetic Retinopathy (NDR) (*n* = 257, 39%), Background Diabetic Retinopathy (BDR) (*n* = 243, 37%), Preproliferative Diabetic Retinopathy (PPDR) (*n* = 135, 21%), and Proliferative Diabetic Retinopathy (PDR) (*n* = 22, 3%); No Diabetic Maculopathy (*n* = 563, 86%) and Diabetic Maculopathy (*n* = 94, 14%). 206 (31%) of the patients had severe vitamin D deficiency with 25(OH)D levels below 10 ng/mL, 284 (43%) were deficient with 25(OH)D of 10–<20 ng/mL, and 101 (14%) were insufficient with 25(OH)D of 20–<30 ng/mL. Only 65 (10%) individuals had “adequate” levels of 25(OH)D at >30 ng/mL. The mean 25(OH)D for the population was 15.8 ± 9.4 ng/mL.

Table 1 shows demographic and metabolic data based on retinopathy grading: NDR, BDR, PPDR, and PDR, respectively. There were no differences in 25(OH)D status between the groups (15.3 ± 9.0 versus 16.4 ± 10.5 versus 15.9 ± 10.4 versus 15.7 ± 8.5, *P* = NS). Subjects were matched for age (59.8 ± 13.8 versus 58.8 ± 13.3 versus 60.8 ± 10.9 versus 55.1 ± 13.6 years); however, the duration of diabetes was significantly lower in NDR (11.3 ± 8.7 versus 18.7 ± 11.7 versus 21.0 ± 9.8 versus 19.7 ± 10.0 years, *P* < 0.0001). The median number of metabolic and anthropometric measurements over the preceding two-year period from the baseline 25(OH)D result was 4 (interquartile range of 3–5). Two-year mean HbA1c (%) (8.2 ± 1.6 versus 8.6 ± 1.7 versus 8.9 ± 1.6 versus 8.9 ± 1.5, *P* < 0.0006) showed a significantly lower HbA1c, lower systolic blood pressure (129 ± 13 versus 131 ± 15 versus 134 ± 15 versus 134 ± 11 mmHg, *P* = 0.007), and higher eGFR (76.3 ± 16.9 versus 75.9 ± 17.5 versus 70.9 ± 16.9 versus 69.0 ± 21.6, *P* = 0.02) in NDR (Table 1). There was no difference for aetiology of diabetes, ethnicity, sex, smoking status, BMI, lipid and bone parameters, and diastolic blood pressure between the grades of DR.

There were no significant differences in the season of assessment in this study (Summer (32%) compared to Spring (22%), Autumn (24%), and Winter (22%)). However, there was a lower level of 25(OH)D in those who had their assessment in Winter (13.7 ± 8.4 ng/mL) compared to Spring (17.3 ± 9.0 ng/mL, *P* = 0.002) and Summer (16.4 ± 10.4 ng/mL, *P* = 0.04) with no difference compared to Autumn (16.0 ± 10.9 ng/mL). Mean value for all seasons was categorised as deficient (10–19.9 ng/mL) and a 3.6 ng/mL difference at most is unlikely to represent any clinical significance.

Table 2 shows logistic regression analyses for DR status with Odds Ratios (OR) and 95% CI. There was no correlation of DR with 25(OH)D (OR 1.00 (95% CI 0.98–1.02), *P* = NS), gender, or ethnicity. However, lower age (OR 0.97 (95% CI 0.96–0.99), *P* = 0.01), longer duration of diabetes (OR 1.09 (95% CI 1.06–1.13), *P* < 0.0001), higher HbA1c (OR 1.22 (95% CI 1.07–1.39), *P* = 0.003), and systolic blood pressure (OR 1.02 (95% CI 1.00–1.04), *P* = 0.02) were all associated with DR.

Table 3 shows demographic and metabolic data in patients with diabetes with (*n* = 94, 14%) and without (*n* = 563, 86%) maculopathy. There were no differences in 25(OH)D status between patients with and without maculopathy (16.2 ± 10.0 versus 15.8 ± 9.8 ng/mL, *P* = NS). Subjects were matched for age (59.1 ± 11.5 versus 59.5 ± 13.3 years); however, the duration of diabetes was significantly longer in patients with maculopathy (15.9 ± 11.1 versus 19.2 ± 9.7 years, *P* = 0.0003). Two-year mean HbA1c (%) (8.4 ± 1.6 versus 9.1 ± 1.5, *P* < 0.0001) and systolic blood pressure (130 ± 14 versus 134 ± 14 mmHg, *P* = 0.01) were significantly higher in patients with diabetes and maculopathy. There were no differences for type of diabetes, ethnicity, sex, smoking status, BMI, lipid and bone parameters, and diastolic blood pressure between patients with and without maculopathy.  Table 4 shows logistic regression analyses for Diabetic Maculopathy status with Odds Ratios and 95% CI. There was no relationship of maculopathy status with 25(OH)D (OR 1.00 (95% CI 0.98–1.03), *P* = NS), age, gender, ethnicity, systolic blood pressure, or lipid fractions. However, a longer duration of diabetes (OR 1.03 (95% CI 1.00–1.05), *P* = 0.01) and higher HbA1c (OR 1.22 (95% CI 1.05–1.43), *P* = 0.009) were associated with maculopathy status.

**Figure 1:**
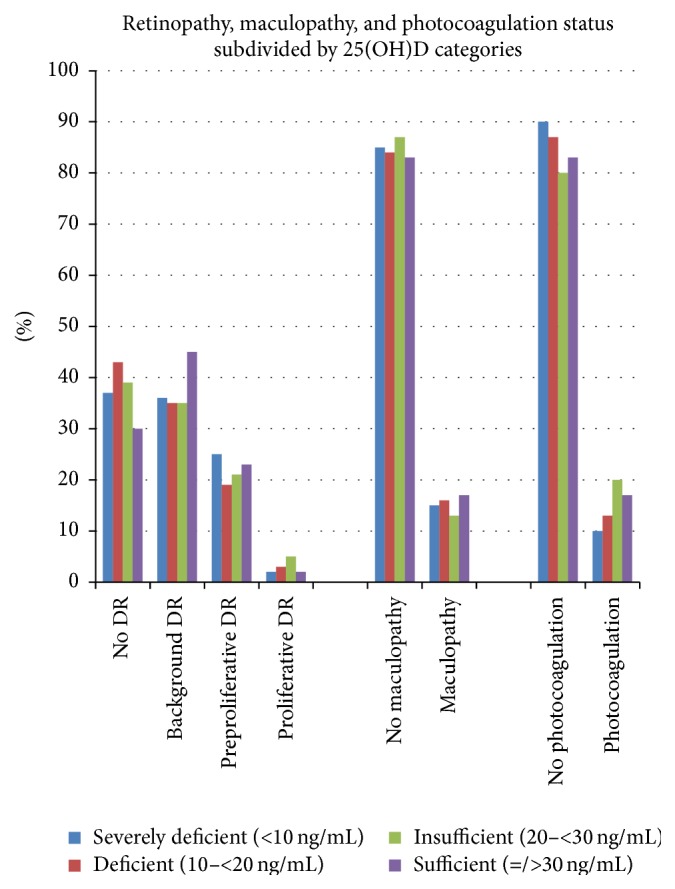
Frequencies of retinopathy, maculopathy, and photocoagulation scarring categorised by 25(OH)D status.

The frequencies for severity of retinopathy, maculopathy, and photocoagulation were similar between the four vitamin D categories (severely deficient (<10 ng/mL), deficient (10–<20 ng/mL), insufficient (20–<30 ng/mL), and sufficient (>30 ng/mL)) (Figure 1).

Categorising the data based on vitamin D status (severely deficient (<10 ng/mL), deficient (10–<20 ng/mL), insufficient (20–<30 ng/mL), and sufficient (>30 ng/mL)), there were no differences in smoking status, sex, total cholesterol, and triglycerides. However, the Chi^2^ analysis for ethnicity was significant (*P* < 0.0001) with a higher proportion of those with severe deficiency (*n* = 189, <10 ng/mL) being of South Asian origin (58%) and those with normal vitamin D status (*n* = 52, >30 ng/mL) being mainly of White European origin (75%). A further Chi^2^ analysis of the aetiology of diabetes was significant (*P* = 0.003) with a greater proportion of type 2 diabetes in the severely deficient group (87%) (<10 ng/mL) compared to those who had a normal vitamin D status (66%) (>30 ng/mL). This reflects our previously published data [29]. BMI was higher in those with severe deficiency (32.1 ± 11.6 kg/m^2^, *P* = 0.02) (<10 ng/mL) and deficiency (31.8 ± 6.7 kg/m^2^, *P* = 0.002) (10–<20 ng/mL) compared to those with adequate vitamin D status (28.6 ± 5.2 kg/m^2^) (>30 ng/mL). Corrected calcium status was marginally higher in those with a normal vitamin D status (2.38 ± 0.12 mmol/L, *P* = 0.02) (>30 ng/mL) compared with those who were deficient (2.33 ± 0.12 mmol/L) (<10 ng/mL) but still within the normal range for serum calcium status.

## 5. Discussion

Vitamin D deficiency has wide ranging implications for insulin resistance, beta cell dysfunction, and hypertension and therefore provides a potential link with diabetic complications. Experimental studies have postulated an important link between vitamin D deficiency and retinopathy [30] and an increased risk of diabetic retinopathy has been demonstrated in the presence of VDR polymorphisms [12].

However, our study has shown no relationship between the vitamin D status and the severity of diabetic retinopathy or maculopathy in a large cohort of patients with predominantly type 2 diabetes, after correcting for glycaemic control, blood pressure, and lipids. We confirm that the “usual culprits” of longer duration of diabetes, higher HbA1c, and systolic blood pressure are directly related to retinopathy and maculopathy [1], thereby providing confidence in the validity of our data. Furthermore, the metabolic and anthropometric measurements used in the regression analysis were taken over an extended period of time as opposed to “spot” readings taken in other studies [23, 25]. A possible explanation for the lack of relationship between vitamin D deficiency and retinopathy could be the striking extent of vitamin D deficiency in this population, although this is consistent with our previous data [29]. Thus, the majority of patients demonstrated deficiency (~90%) and indeed severe deficiency (~31%). Therefore, any relationship between retinopathy and adequacy of vitamin D could not be explored adequately. Furthermore, there were only a small number of patients with Diabetic Maculopathy (*n* = 94, 14%) in this study, which ultimately limits the power of the analysis. Only a limited number of clinical studies have investigated the role of vitamin D deficiency in DR. In one of the earliest studies, Aksoy et al. showed an inverse relationship between 1,25(OH)_2_D_3_ and worsening retinopathy, although the short half-life of 1,25(OH)_2_D_3_ may limit the interpretation of any such relationship [23]. Another smaller North American study has shown that subjects with DR, in particular PDR, have lower levels of 25(OH)D [25]. Whilst in a recent study the percentage of individuals with vitamin D deficiency increased with the severity of retinopathy, regression analysis did not demonstrate a statistically significant relationship between retinopathy severity and serum 25(OH)D concentration [26]. In a prospective observational follow-up study of a cohort of patients with type 1 diabetes, although severe vitamin D deficiency independently predicted all-cause mortality, it was not related to the development of either retinopathy or nephropathy [4]. In a recently published study of 715 patients with type 2 diabetes, serum 25(OH)D levels decreased significantly in relation to the severity of either retinopathy or nephropathy or both [31]. However, in the prospective EURODIAB study conducted in subjects with type 1 diabetes, both higher 25(OH)D_2_ and 25(OH)D_3_ were associated with a lower prevalence of macroalbuminuria, but not retinopathy and cardiovascular disease [32].

This large cross-sectional study found no association of vitamin D status with diabetic retinopathy or maculopathy. A population with a larger spread of vitamin D levels may provide further insight into a possible association, but this may not be possible due to the high prevalence of vitamin D deficiency. In the long term, randomised controlled trials of adequate vitamin D intervention and diabetic microvascular outcomes are required to truly assess any potential therapeutic benefit.

## Figures and Tables

**Table 1:** Demographic and metabolic parameters in subgroups based on severity of retinopathy.

	No Diabetic Retinopathy (NDR) (*n* = 257)	Background Diabetic Retinopathy (BDR) (*n* = 243)	Preproliferative Diabetic Retinopathy (PPDR) (*n* = 135)	Proliferative Diabetic Retinopathy (PDR) (*n* = 22)	*P* value
Age (years)	59.8 ± 13.8	58.8 ± 13.3	60.8 ± 10.9	55.1 ± 13.6	NS
Duration of diabetes (years)	11.3 ± 8.7^†^	18.7 ± 11.7^†^	21.0 ± 9.8^†^	19.7 ± 10.0^†^	**<0.0001**
Type 2 DM (%)	88	75	80	77	NS
Ethnicity (White European/South Asian (%))	48/45	55/38	50/45	52/48	NS
Sex (male (%))	51	50	48	53	NS
Current smokers/past smokers/never smoked (%)	14/25/61	14/33/53	14/26/60	4/23/71	NS
BMI (kg/m^2^)	31.6 ± 10.6	31.1 ± 7.1	31.9 ± 6.4	31.1 ± 6.9	NS
25(OH)D (ng/mL)	15.3 ± 9.0	16.4 ± 10.5	15.9 ± 10.4	15.7 ± 8.5	NS
HbA1c (%)	8.2 ± 1.6^*∗*^	8.6 ± 1.7^*∗*^	8.9 ± 1.6^*∗*^	8.9 ± 1.5	**<0.0001**
TC (mmol/L)	4.1 ± 1.0	4.2 ± 1.1	4.2 ± 1.3	4.3 ± 1.1	NS
HDL (mmol/L)	1.3 ± 0.5	1.4 ± 0.5	1.4 ± 0.7	1.3 ± 0.4	NS
Trig (mmol/L)	1.8 ± 0.9	1.8 ± 1.5	1.8 ± 0.9	2.5 ± 2.9	NS
eGFR (mL/min/L)	76.3 ± 16.9^*∗∗*^	75.9 ± 17.5	70.9 ± 16.9^*∗∗*^	69 ± 21.6	**0.02**
Systolic BP (mmHg)	129 ± 13^*∗∗∗*^	131 ± 14.6	134 ± 15^*∗∗∗*^	134 ± 11	**0.007**
Diastolic BP (mmHg)	70 ± 7	69 ± 7	70 ± 8	71 ± 8	NS
ALP (u/L)	87 ± 39	83 ± 34	84 ± 32	93 ± 43	NS
CCa (mmol/L)	2.4 ± 0.1	2.4 ± 0.1	2.3 ± 0.1	2.4 ± 0.2	NS

Duration of diabetes: ^†^NDR versus BDR (*P* < 0.0001) versus PPDR (*P* < 0.0001) versus PDR (*P* = 0.001).

HbA1c: ^*∗*^NDR versus BDR (*P* = 0.01) versus PPDR (*P* < 0.0001).

eGFR: ^*∗∗*^NDR versus PPDR (*P* = 0.04).

Systolic BP: ^*∗∗∗*^NDR versus PPDR (*P* = 0.008).

BMI: Body Mass Index, BP: blood pressure, TC: total cholesterol, HDL: high-density lipoprotein cholesterol, Trig: triglycerides, ALP; alkaline phosphatase, CCa; corrected calcium, and eGFR: estimated glomerular filtration rate.

**Table 2:** Logistic regression analyses for the relationship between retinopathy, 25(OH)D status, and other confounding variables.

	Odds Ratio	95% CI	*P*
25(OH)D	1.00	0.98–1.02	NS
Age (years)	**0.97**	**0.96–0.99**	**0.01**
Duration of diabetes (years)	**1.09**	**1.06–1.13**	**<0.0001**
Never smoked	0.48	0.21–1.09	NS
HbA1c (%)	**1.22**	**1.07–1.39**	**0.003**
TC (mmol/L)	1.09	0.88–1.36	NS
HDL (mmol/L)	0.88	0.55–1.41	NS
Triglycerides (mmol/L)	0.98	0.77–1.25	NS
Systolic BP (mmHg)	**1.02**	**1.00–1.04**	**0.02**
Diastolic BP (mmHg)	0.98	0.94–1.01	NS
eGFR (mL/min/L)	0.99	0.98–1.00	NS

BMI: Body Mass Index, BP: blood pressure, TC: total cholesterol, HDL: high-density lipoprotein cholesterol, ALP: alkaline phosphatase, CCa: corrected calcium, and eGFR: estimated glomerular filtration rate.

**Table 3:** Demographic and metabolic parameters in subgroups based on maculopathy.

	No Diabetic Maculopathy (*n* = 563)	Diabetic Maculopathy (*n* = 94)	*P*
Age (years)	59.5 ± 13.3	59.1 ± 11.5	NS
Duration of diabetes (years)	15.9 ± 11.1	19.2 ± 9.7	**0.0003**
Type 2 DM (%)	82	80	NS
Ethnicity (White European/South Asian (%))	40/50	49/48	NS
Sex (male (%))	51	47	NS
Current smokers/past smokers/never smoked (%)	15/28/57	16/27/57	NS
BMI	31.4 ± 8.8	31.3 ± 6.2	NS
25(OH)D (ng/mL)	15.8 ± 9.8	16.2 ± 10.0	NS
HbA1c (%)	8.4 ± 1.6	9.1 ± 1.5	**<0.0001**
TC (mmol/L)	4.1 ± 1.1	4.4 ± 1.4	NS
HDL (mmol/L)	1.3 ± 0.5	1.4 ± 0.8	NS
Trig (mmol/L)	1.8 ± 1.2	1.9 ± 1.6	NS
eGFR	75 ± 18	73 ± 20	NS
Systolic BP (mmHg)	130 ± 14	134 ± 14	**0.01**
Diastolic BP (mmHg)	71 ± 7	70 ± 7	NS
ALP (u/L)	87 ± 53	100 ± 129	NS
CCa (mmol/L)	2.3 ± 0.1	2.4 ± 0.1	NS

BMI: Body Mass Index, BP: blood pressure, TC: total cholesterol, HDL: high-density lipoprotein cholesterol, Trig: triglycerides, ALP: alkaline phosphatase, CCa: corrected calcium, and eGFR: estimated glomerular filtration rate.

**Table 4:** Logistic regression analyses for the relationship between maculopathy, 25(OH)D status, and other confounding variables.

	OR	95% CI	*P*
25(OH)D	1.00	0.98–1.03	NS
Age (years)	0.99	0.97–1.02	NS
Duration of diabetes (years)	**1.03**	**1.00–1.05**	**0.01**
Never smoked	1.24	0.73–2.12	NS
HbA1c (%)	**1.22**	**1.05–1.43**	**0.009**
TC (mmol/L)	1.05	0.84–1.33	NS
HDL (mmol/L)	1.12	0.73–1.73	NS
Triglycerides (mmol/L)	1.19	0.77–1.25	NS
Systolic BP (mmHg)	1.01	0.99–1.04	NS
Diastolic BP (mmHg)	1.03	0.98–1.07	NS
eGFR (mL/min/L)	0.99	0.97–1.00	NS

BMI: Body Mass Index, BP: blood pressure, TC: total cholesterol, HDL: high-density lipoprotein cholesterol, ALP: alkaline phosphatase, CCa: corrected calcium, and eGFR: estimated glomerular filtration rate.
